# Hybrid of Smith-Peterson and Watson-Jones minimally invasive direct anterior approach to the hip joint

**DOI:** 10.1186/s12891-023-06254-8

**Published:** 2023-02-28

**Authors:** Ting Zhang, Sambhu Choudhury

**Affiliations:** 1 Mercy Health–Cincinnati Sports Medicine and Orthopaedic Center, Cincinnati Sports Medicine Research and Education Foundation, Cincinnati, OH USA; 2 Cincinnati VA Medical Center, Cincinnati, OH USA

**Keywords:** Direct anterior approach, Hueter interval, ABEL approach, Minimally invasive, Hip replacement

## Abstract

**Background:**

Muscle-sparing techniques, more consistent acetabular component positioning with fluoroscopy guidance, development in implants and instrumentation, expedited rehabilitation, and patients’ expectations have led to increased utilization of various direct anterior and anterolateral approaches to the hip joint.

**Methods and surgical technique:**

In this technical note, we demonstrate for the first time a hybrid modification of traditional Smith-Peterson and Watson-Jones approaches to the hip joint on a standard operating room (OR) table.

**Conclusions:**

As demonstrated in this article, a precise knowledge of anatomy and clear goals in the surgical approach can minimize complications and facilitate visualization and instrumentation placement in the “direct anterior approach” to the hip joint.

## Background

Muscle-sparing, more consistent acetabular component positioning with fluoroscopy guidance, development in implants and instrumentation, expedited rehabilitation, and patients’ expectations have led to increased utilization of various direct anterior and anterolateral approaches to the hip joint [1–3]. Aging population expect rapid recovery, minimal invasiveness, and higher functions with anterior muscle-sparing approaches. Pioneer surgeons Carl Hueter, Marius Smith-Peterson, and multiple other authors described the direct anterior approach to the hip which consistently utilizes the muscular interval between the sartorius and the tensor fasciae latae (TFL) muscle, which is known as the Hueter interval [4]. One limitation of this approach is the risk of damaging the lateral femoral cutaneous nerve (LFCN) as its main trunk usually runs along the medial border of the proximal TFL muscle. To minimize the risk for iatrogenic nerve injury during the approach, some authors advocated a modification where they incise the superficial thigh fascia as laterally as possible over the belly of the TFL followed by blunt dissection between the muscle and the superficial fascia, retracting TFL muscle belly laterally and entering the Hueter interval [5]. The TFL and gluteus medius surgical interval was first described by Dr. Louis Sayre in 1854, treating a sequela of septic arthritis in a 9-year-old patient [4]. Sir Reginald Watson-Jones further developed Sayre’s approach in the 1930’s to the classic “Watson-Jones” approach with a detachment of the anterior 1/3 of abductors from the anterior facet of the greater trochanter [4]. In the early 2000s, Dr. Heinz Rottinger described the ABLE Advanced anterior Approach using the TFL and gluteus medius interval in a soft tissue-sparing fashion without abductor detachment while the patient positioned lateral decubitus position using peg boards [3]. In this technical note, we describe a minimally invasive soft tissue-sparing anterior approach to the hip where we incise the superficial thigh fascia as laterally as possible in protecting the LFCN with our superficial dissection, but instead of retracting the TFL laterally and exploring the Hueter interval, we bluntly develop an interval between TFL muscle belly and superficial fascia laterally, retracting TFL medically, hence, essentially exploring the muscle interval between TFL and gluteus medius without detaching any abductors from the greater trochanter in a supine position on a standard table. Maccagnano et al. showed that direct anterior approach had lower blood loss, faster surgical time compared to direct lateral approach treating patients suffering femoral neck fractures during the COVID-19 pandemic [6]. Our aim is to present a safe and reliable approach that combines the advantages of the traditional direct anterior and anterolateral approach but avoids their pitfalls. Additionally, we aim to present a cost-effective, safe, and easy positioning of the patient on a simple standard table in a supine position to perform a complete muscle-sparing anterior approach to the hip joint.

### Surgical technique

#### Step I: positioning

General anesthesia is administered, and the patient is positioned supine on a standard table flipped with the patient’s head positioned at the foot part of the table. There are two advantages positioning patient’s head on the foot part of the standard OR table: 1. When the surgeon instruments the proximal femur, if we need to break the table to allow hip extension, the break of the table is at patient’s hip level instead of at the knee; 2. By positioning patient’s head over the foot part of the table, it allows room for C-arm to come in for intra-operative fluoroscopy. An additional arm board is connected to the side of the non-operative leg to allow further abduction as needed during the case.

#### Step II: skin incision

We identify the anterior iliac crest and the anterior superior iliac spine (ASIS). We then mark our incision on the imaginary line from ASIS to the ipsilateral fibula head. The incision typically starts two centimeters lateral and distal from the ASIS, approximately 8 cm in length on average.

#### Step III: superficial dissection (Fig. 1)

We dissect the superficial subcutaneous fat layer with monopolar coagulation and hemostasis. The deep subcutaneous fat overlying the superficial thigh fascia is typically swept from the fascia gently with a surgical sterile gauze to prevent iatrogenic injuries to the LFCN, accidentally entering the superficial fascia, and to aid in the identification of the TFL perforator vessel to ensure an accurate anatomic location to enter the superficial fascia [7].

**Fig. 1:**
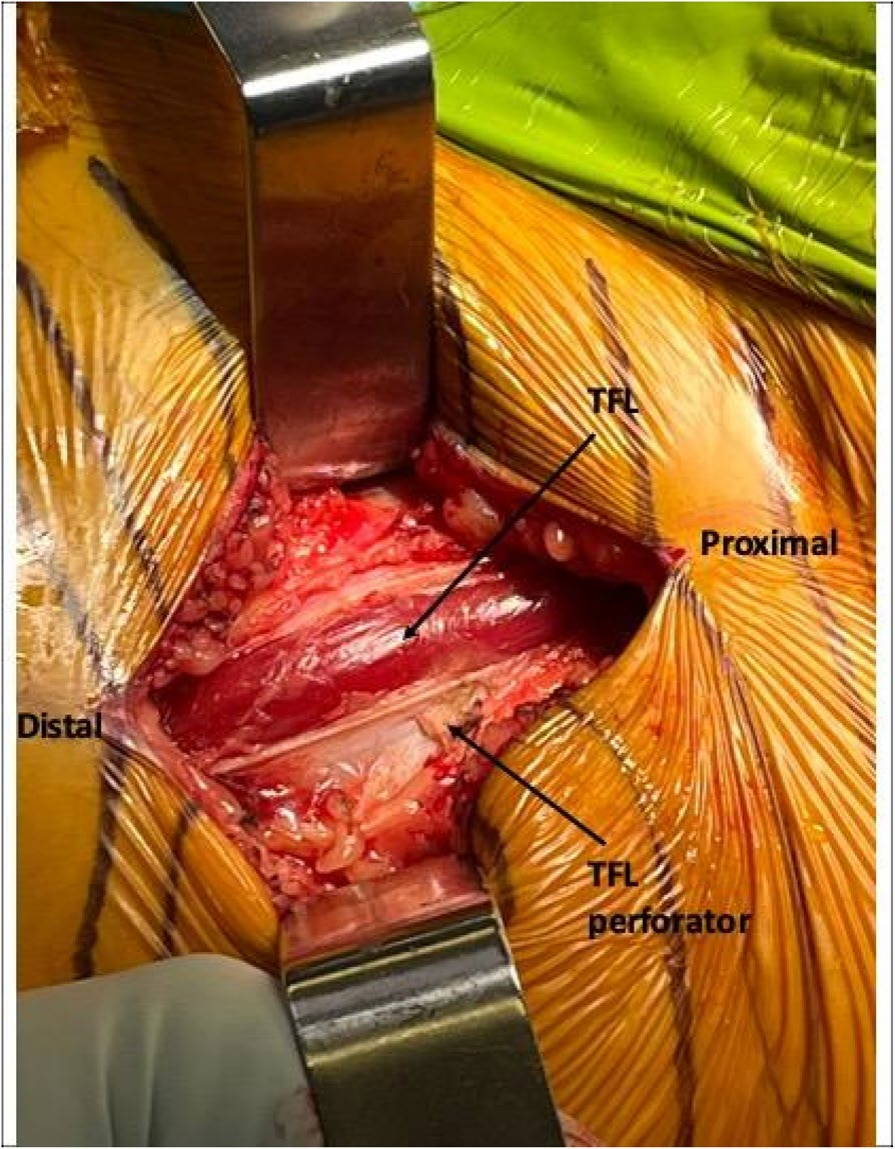
Superficial Dissection

#### Step IV: deep dissection (Figs. 2A,B,C,D; and 3)

We typically use a Cobb elevator to gently peel the TFL muscle belly away from the superficial fascia laterally, exploring the interval between TFL and gluteus medius. Care should be taken here to coagulate the perforating vessels laterally between the TFL muscle belly and its investing fascia. Once we have the TFL muscle belly retracted medially and hemostasis achieved, we put a blunt Homann retractor laterally in the saddle area of the superior femoral neck, retracting the gluteus medius muscle laterally. We then use Aquamantys (Medtronic, Warsaw, IN) both as a dissection tool and as a great tool for hemostasis as we coagulate the circumflex vessels overlying the anterior femoral neck crossing the surgical field as we encounter them and gently dissect muscles off the anterior femoral neck. We then identify the medial femoral neck and place another blunt Homann retractor just proximal to the lesser trochanter. We also place a sharp Homman retractor proximally on the anterior acetabulum, retracting towards the contralateral shoulder which helps us identify the entirety of the anterior capsule.

**Fig. 2:**
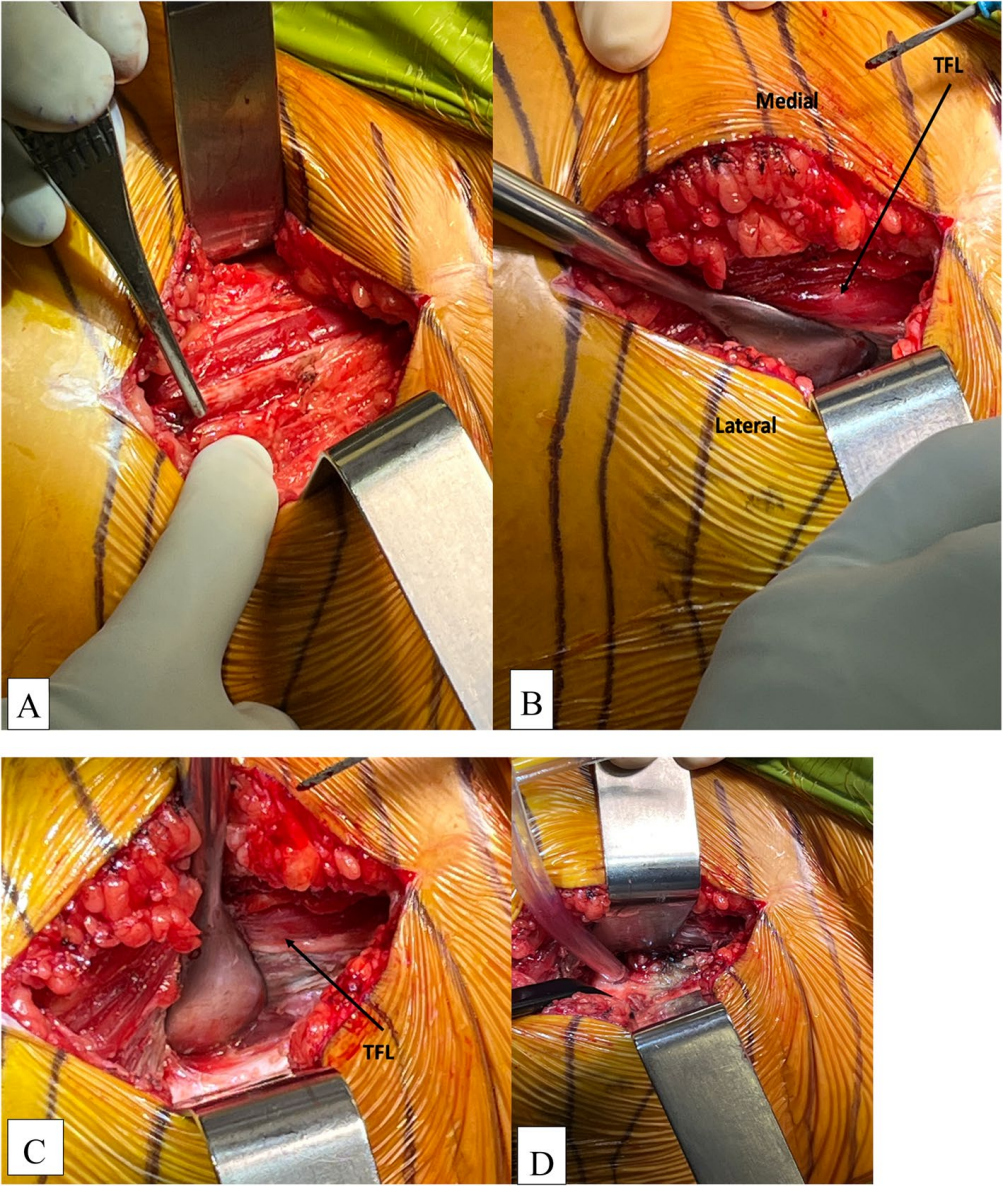
Peeling TFL lateral to medial to expose the deep TFL fascia using a Cobb elevator. **A** Pick up is on TFL fascia **B** Cobb elevating TFL muscle medially **C** Cobb elevating TFL muscle medially **D** Make sure to coagulate perforating branches laterally

**Fig. 3:**
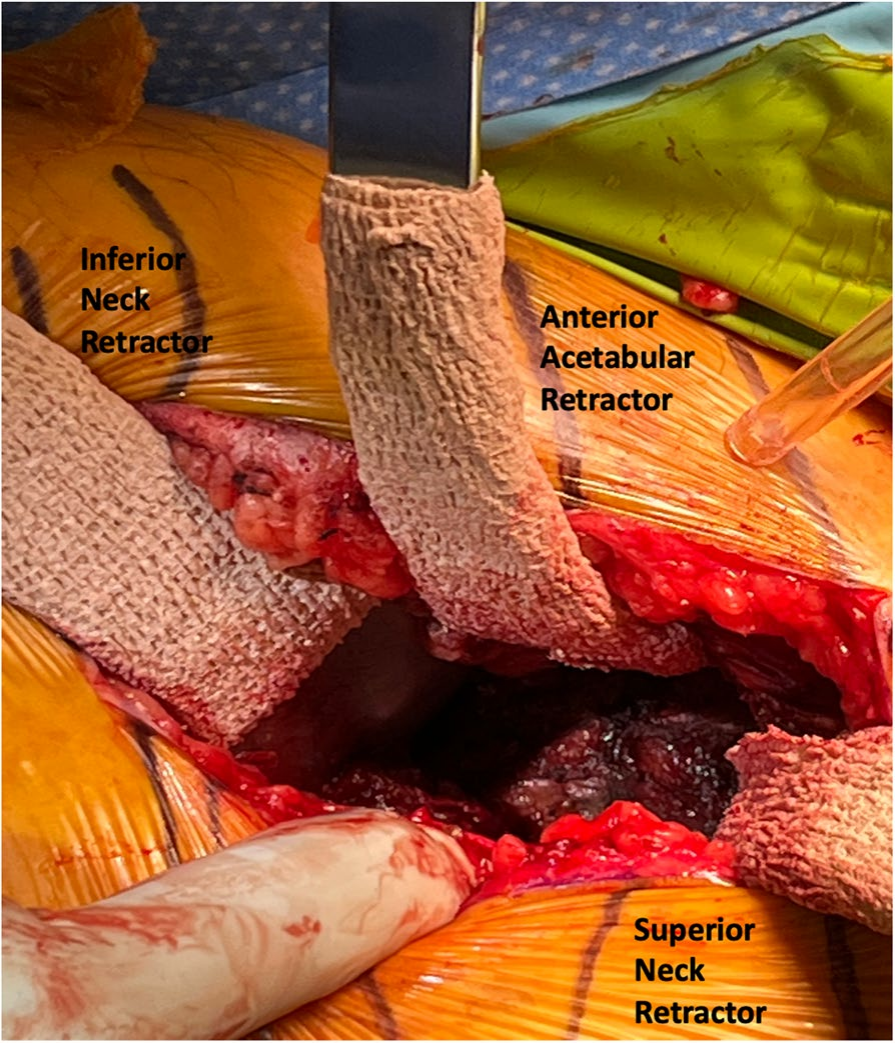
Defining femoral head and neck extracapsular

#### Step V: capsulotomy and initial soft tissue releases (Figs. 4, 5 and 6)

We use a combination of Cobb elevator and Rongeur to clear the fat/soft tissue overlying the anterior capsule, then a meticulous T-Capsulotomy is performed with monopolar electrocautery. We repair this robust anterior tissue at the end of the case for enhanced anterior stability; hence, meticulous capsulotomy is elected instead of capsulectomy. Once the hip joint is entered, we reposition the Homann retractors inside the joint capsule directly against bone and use the retractors to create tension, aiding in necessary soft tissue releases to ensure exposure and instrumentation of the acetabulum as well as the proximal femur in a systematic and stepwise fashion, specifically releasing the anterior joint capsule. We always preserve the abductor tendon, and no additional extra-capsular soft tissue are released at this step. We then use an osteotome to take out the anterior osteophyte to aid in femoral head extraction after the femoral neck cut. We also create a “napkin ring” femoral neck cut to aid in femoral head extraction.

**Fig. 4:**
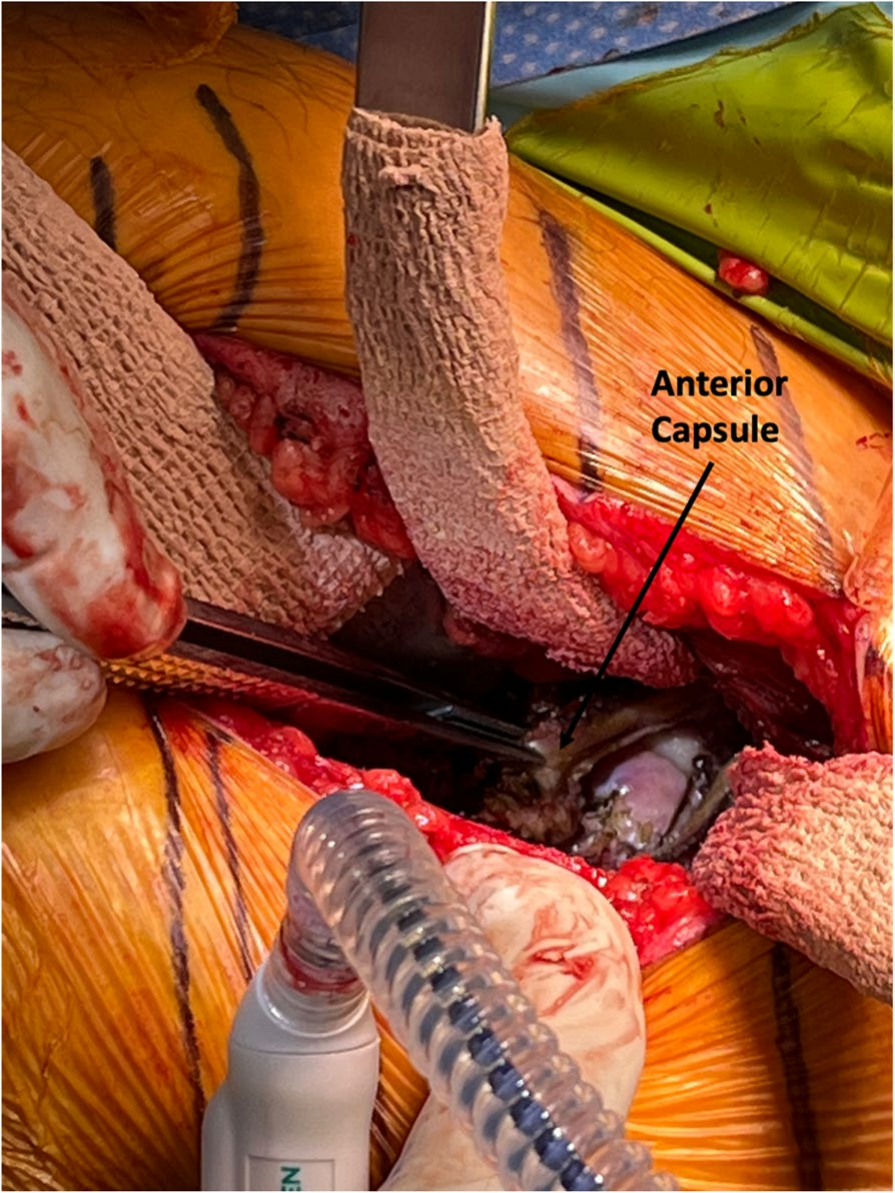
Capsulotomy

**Fig. 5:**
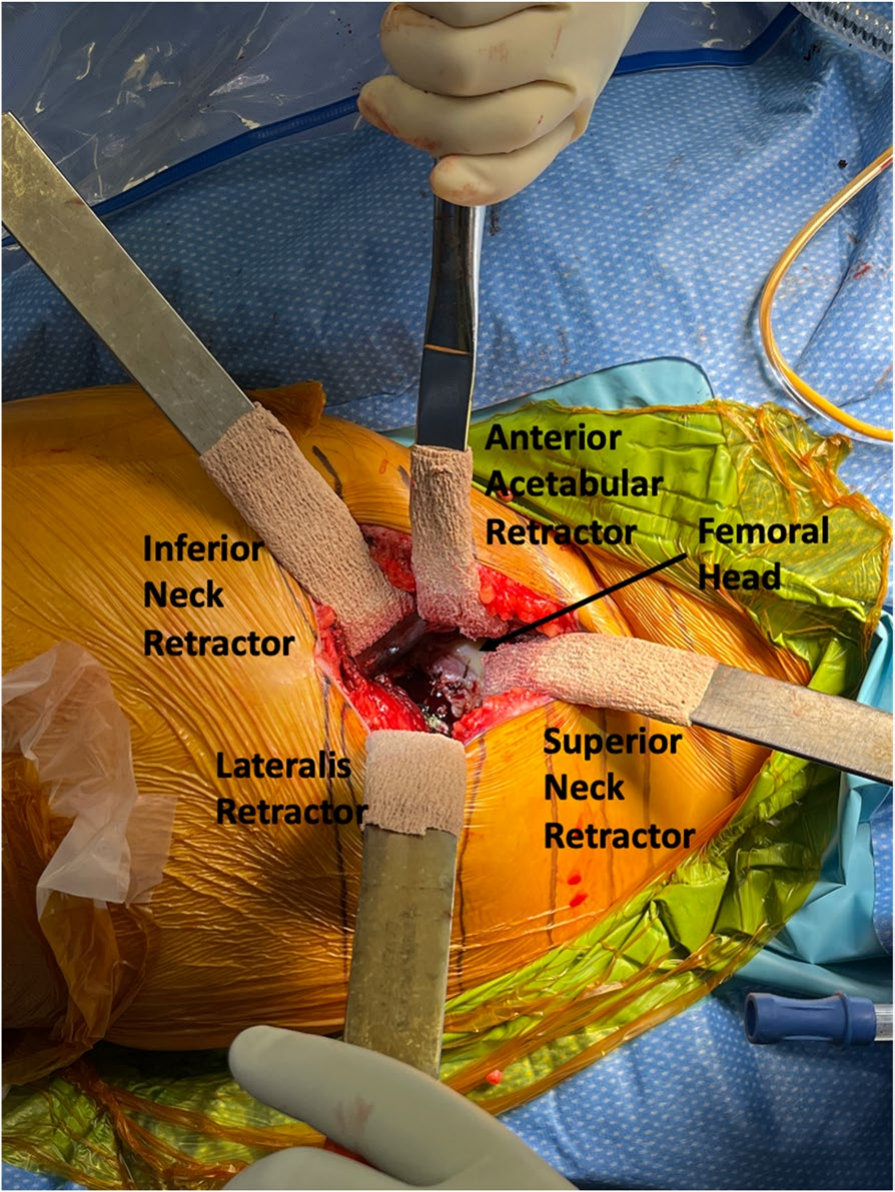
Retractor placements

**Fig. 6:**
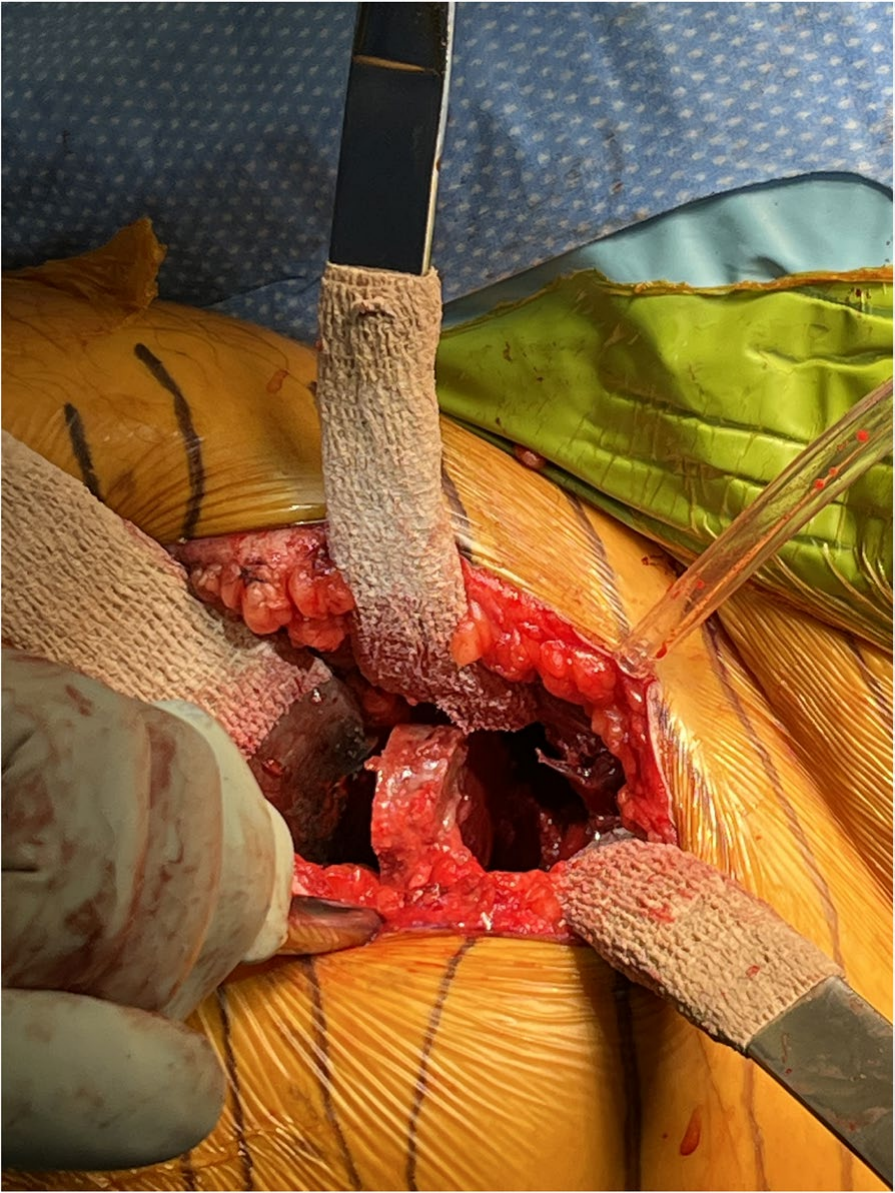
Napkin ring cut of femoral neck to assist in head extraction

#### Step VI: acetabular preparation with fluoroscopy guidance (Fig. 7)

We place two retractors, one at the 3 o’clock position and one at the 9 o’clock position for acetabular preparation. First, we remove the labrum circumferentially and purposefully leave the pulvinar tissue in the cotyloid fossa as the senior author of this paper found that leaving the soft tissue does not impede his ability to ream or impact the final component but is potentially beneficial from hemostasis and visualization standpoint. Aggressive removal of pulvinar tissue can lead to bleeding in the cotyloid fossa which impedes visualization of the medial wall of the acetabulum. Then the acetabulum is reamed in the usual standard fashion with the final component impacted under fluoroscopy guidance. We prefer to place two screws in the safe zone for additional fixation.

**Fig. 7:**
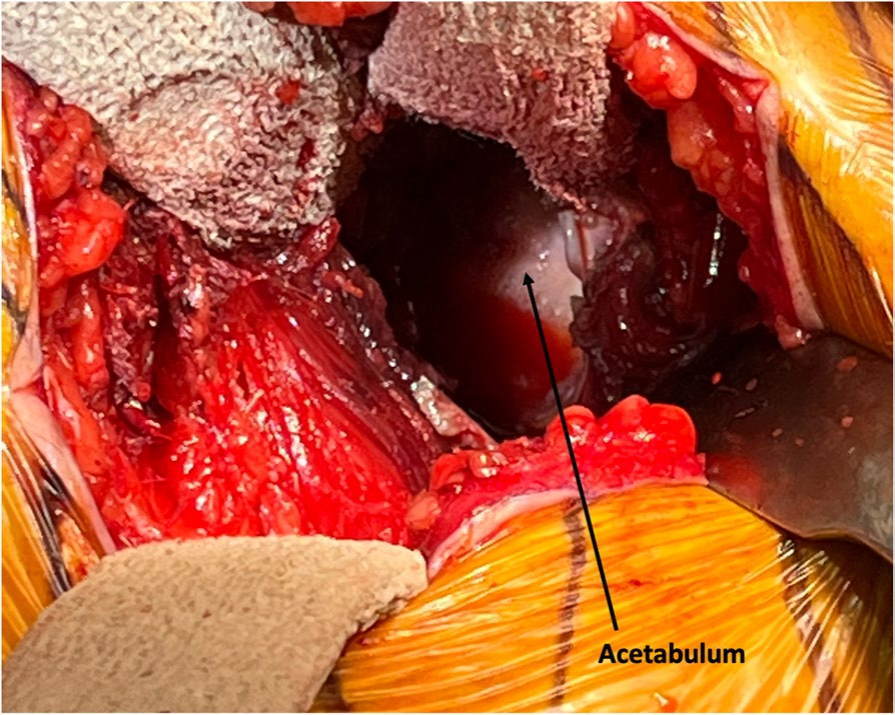
Acetabular exposure with retractor placements

#### Step VII: systematic proximal femur release (Fig. 8)

We abduct the non-operative leg as needed onto the additional arm board connected at the beginning of the case. Then operative extremity is externally rotated and adducted, placing a Cobra retractor in the bare area of the greater trochanter, protecting the abductor muscles and tendons. A bone hook is used intramedullary at the proximal neck to pull the proximal femur anterolaterally. Additional releases along the proximal femoral neck are performed to ensure safe instrumentation.

**Fig. 8:**
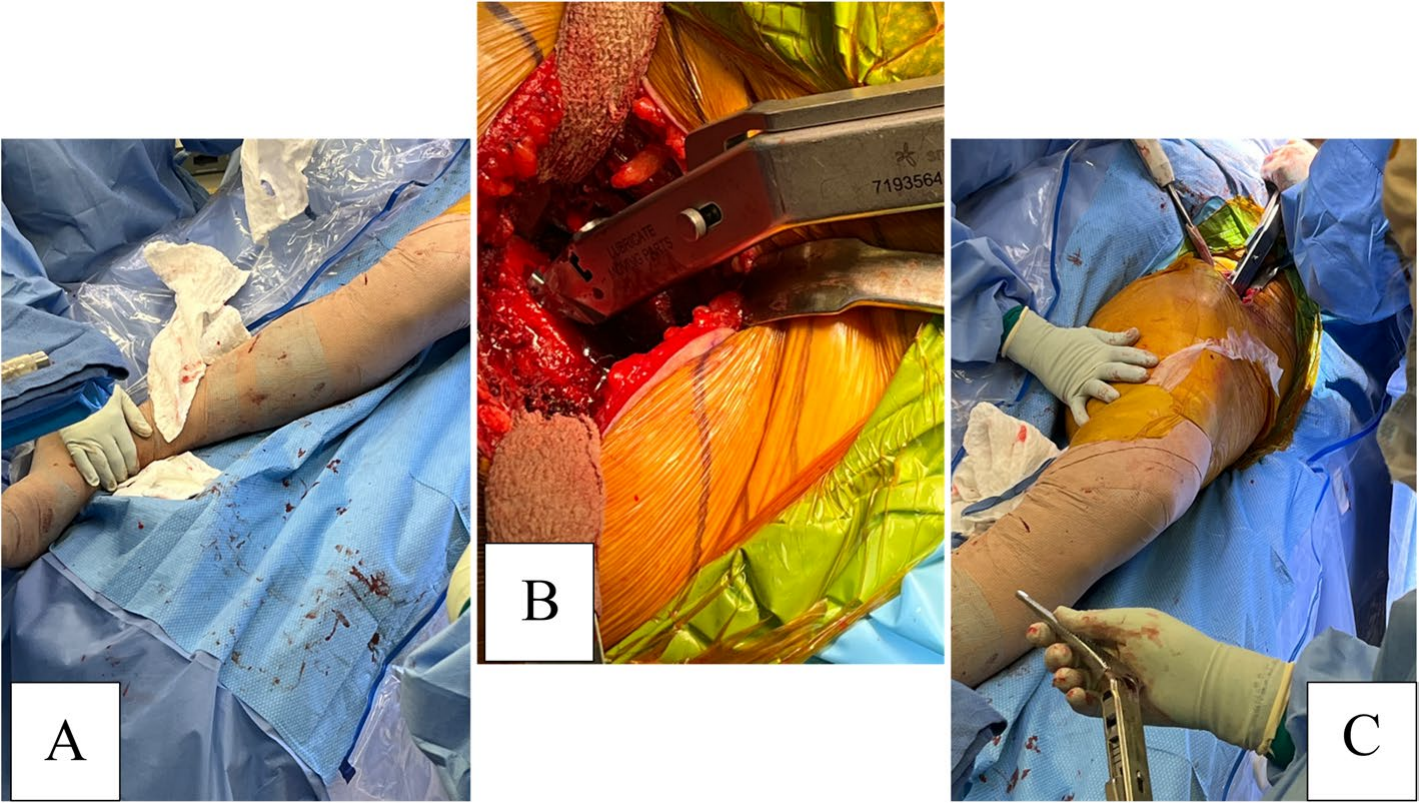
Femoral instrumentation **A** Showing adduction and external rotation of the limb **B** Exposure and broaching of proximal femur **C** Birdseye view of the lower limb during instrumentation

#### Step VIII: femoral instrumentation (Figs. 8 and 9)

Box osteotome is used to enter the femoral canal. Lateralizing rasp is used to prevent varus instrumentation. Sequential manual broaching is used. Trendelenberg of the table is used to extend the operative leg 30–40 deg as needed. Once the trial stem and neck are selected, the table is leveled, standard reduction maneuver is performed to reduce the hip joint. Shuck test, anterior and posterior stability throughout the range of motion is checked on the table. Then, fluoroscopy is brought in to assess offset and leg length. Appropriate adjustments are made accordingly, then definitive femoral components are impacted.

**Fig. 9:**
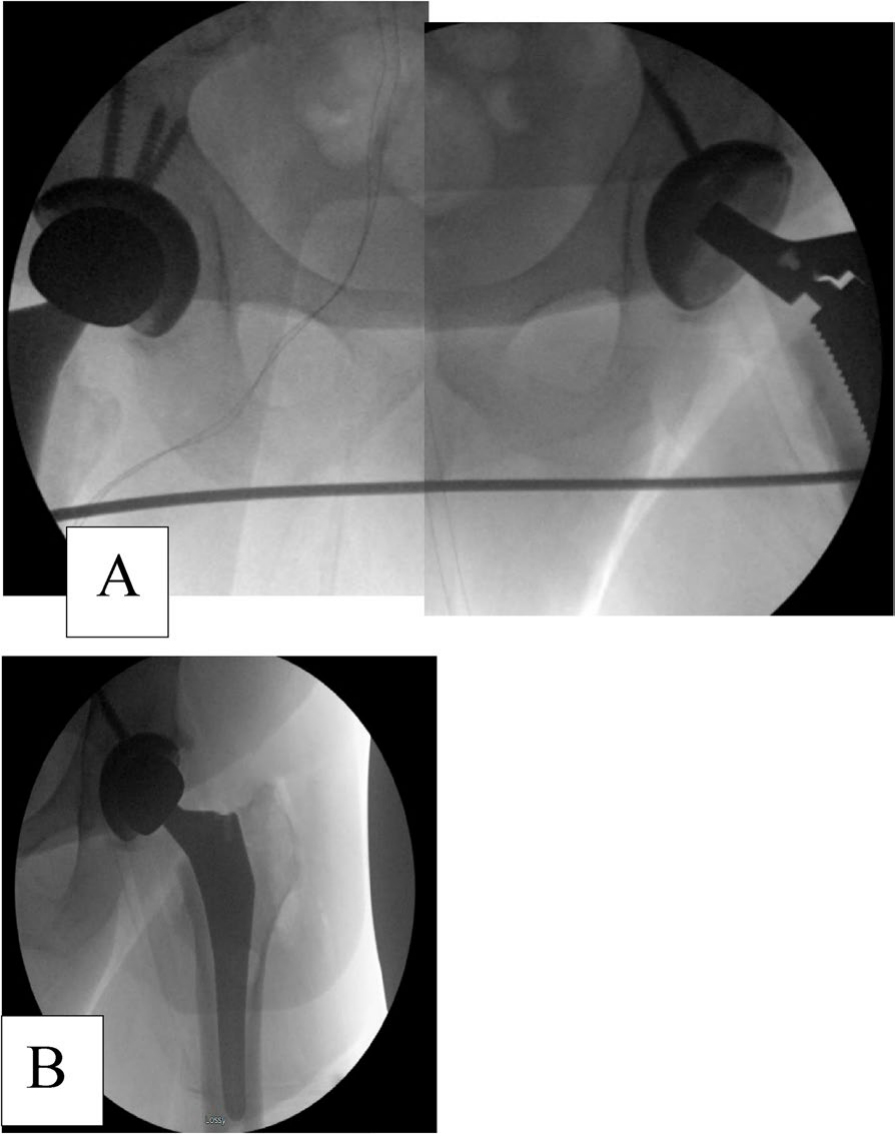
Fluoroscopy image to assess length and offset **A** Showing equal leg length with fluoroscopy **B** Showing final components of the replaced hip

#### Step VIIII: capsule closure (Fig. 10)

As mentioned, previously, we do use non-absorbable braided Ethibond sutures to reapproximate the anterior capsule and close this robust tissue overlying the implant for aided stability and prevention of deep infection.

**Fig. 10:**
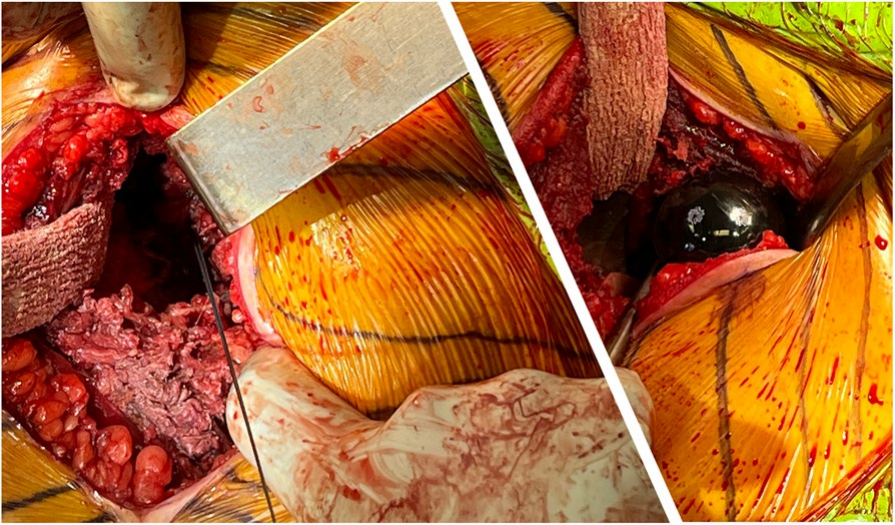
Final component and capsular closure

The surgical wound is irrigated and closed in the usual standard fashion.

### Pearls and pitfalls

#### Pearls

Standard table, supine positioning, additional arm board.

Protection of LFCN.

Exploration of the interval between TFL and gluteus medius without detaching the anterior 1/3 of the abductor.

#### Pitfalls

Use of Hana table, torque on operative knee and ankle, no direct surgeon control, limited one-directional stability assessment at the end of the case.

Not able to find the correct interval.

Damage to LFCN.

Detaching anterior abductors.

Inconsistent/inadequate proximal femoral release causing difficulties in exposure and instrumentation.

## Discussion

The traditional direct anterior approach to the hip joint sometimes creates exposure difficulties even for experienced surgeons due to muscular TFL being retracted laterally [2]. Many training programs grade their junior surgeons and trainees on the health of TFL muscle at the end of the case to assess mastery and competence doing this approach and procedure. On the other hand, exploring the interval between TFL and abductors does create a more direct path and better exposures of the proximal femur as well as the acetabulum as surgeons no longer need to “fight against” the big muscular TFL laterally for the entire case. The learning curve of this variant surgical approach is like the traditional direct anterior and anterolateral approaches. The added benefit of this approach is allowing surgeons to perform this approach on a standard OR table instead of a HANA table mitigating the need to hyper-extend the hip to deliver the proximal femur to the surgical window for instrumentation. From cost-saving standpoint, standard OR table is cheaper compared to HANA table. Additionally, it is easier for the surgical crew to position patient supine on a standard OR table vs. HANA table. The traditional Watson-Jones approach describes detachment of the anterior 1/3 abductors [1]. Doing our approach ameliorate this step and reduce any damage to the abductors. The senior author of this technique noted had difficulties reliably identifying the interval between TFL and abductors as originally described in the ABEL approach [3]. Doing the approach as we described increases the reliability and consistency in exploring the correct interval at the beginning of the case.

## Conclusion

Here we presented a safe and reliable approach that combined the advantages of the traditional direct anterior and anterolateral approach but avoided their pitfalls. Additionally, we presented a cost-effective, safe, and easy positioning of the patient on a simple standard table in a supine position to perform a complete muscle-sparing anterior approach to the hip joint.

## Data Availability

All the material is owned by the authors and/or no permissions are required. We described and published our original surgical technique. Data can be made available from Sambhu Choudhury and Ting Zhang.
